# A Case of Cruro-Scrotal Hernia, with the Testis in the Perineum

**Published:** 1901-03-09

**Authors:** Eccles McAdam

**Affiliations:** Assistant Surgeon, City of London Truss Society and West London Hospital; Examiner in Anatomy to the Society of Apothecaries, &c.


### HOSPITAL CLINICS AND MEDICAL PROGRESS.

### A CASE OF CRURO-SCROTAL HERNIA, WITH THE TESTIS IN THE PERINEUM.

RADICAL OPERATION UPON THE HERNIA, AND TRANSPLANTATION OF THE TESTIS INTO THE SCROTUM.

By W. McADAM ECCLES, M.S., F.R.C.S., Assistant Surgeon, City of London Truss Society and West London Hospital; Examiner in Anatomy to the Society of Apothecaries, &c.

A MAN, aged forty, was admitted into the West London Hospital having been sent to me by Dr. Best, of Waltham Cross, on account of hernia and displacement of the testis. By occupation he was a stableman, and had been married for some years, but without children. A swelling had been noticed in the right scrotal region for the past ten years, and had on several occasions given rise to much discomfort, with now and again symptoms of strangulation, requiring medical aid. He had worn a truss during these years, which, however, had not satisfactorily controlled the protusion. He is said to have been subject to cough for some time, but otherwise he is a healthy man. On examination the case was found to be one in which the right testis, having missed the entrance into the scrotum, had passed backwards into the perineum. A hernia had descended into a patent processus vaginalis into the cruro-scrotal fold, and had accompanied the testis so far on its way into its abnormal position. The right half of the scrotum was well developed, and the dartos active. The operation advised had two objects in view: one the cure of the hernia, and the other, transplantation of the testis into the scrotum. Often in cases of congenital displacement of the testis there is some want of proper development of the muscles of the lower part of the abdominal wall, and this fact makes the result of a radical operation upon the hernia not so certain as in the more usual forms of congenital inguinal hernia. A testis which has migrated into the perineum is frequently one that is fairly well developed, and is therefore fully worthy of transplantation into the scrotum. In a patient of this age the transference is of course not with a view to any further growth of the gland, but rather to place it in such a position that it may be less in the way of danger.

In operating upon the case, the primary incision is to be made, not over the site of the testis, but, as is usual for herniotomy, over the line of the inguinal canal. This was carried out in this case, and the hernial sac exposed and removed, flush with the parietal peritoneum, the lower part of the sac being left to form a tunica vaginalis for the testis. The gland was drawn up into the inguinal region so as to appear in the wound, and then after freeing its attachments to the tissues around, a bed was made for it by means of the finger and forceps in the scrotal tissues.

It was transplanted thither, and held in position by means of a silk thread passed through the lower end of the organ, and tied over a small roll of gauze at the lowest part of the scrotum. Owing to the length of the spermatic cord, there was no difficulty in placing the testis in its new quarters. By this method of transplantation a substantial mass of tissue is left between the old and the new position of the testis—a matter of considerable importance in the prevention of a return of the gland into its abnormal site. After the transference of the testis, the inguinal canal was sutured by silk stitches carried through the margin of the arching fibres of the internal oblique and transversalis muscles and the posterior margin of Poupart's ligament, the spermatic cord being left in the canal.

Finally, the aponeurosis of the external oblique was sutured with interrupted stitches of silk, and the skin and subcutaneous tissues brought together with silkworm gut. By this means the three tiers of sutures are not at the same level, the deepest being the lowest, and the most superficial being the highest. It is thus that a greater strength is imparted to the inguinal region. The parts were dressed with a gauze and collodion dressing, which was left undisturbed until the eighth day, when it was removed and the superficial sutures cut out. The wound healed by primary union, and the testis, even after being released from the suture that held it in the scrotum, showed no tendency to slip back into its old quarters. The patient left the hospital at the end of three weeks, and a truss was applied.

